# Prevention of postpartum suicidality in Israel

**DOI:** 10.1186/s13584-019-0347-z

**Published:** 2019-10-28

**Authors:** Anat Brunstein Klomek

**Affiliations:** Baruch Ivcher School of Psychology, Interdiciplinary Center (IDC), PO BOX 167, 8 Ha university Street, 46101 Herzliya, Israel

**Keywords:** Postpartum suicide, Suicidal ideation, Postpartum depression, Israel

## Abstract

Postpartum suicidality in Israel had not been systematically studied until the recent important investigation by Glasser and colleagues. The authors review rates, trends, and characteristics of postpartum women who considered, attempted, or completed suicide in Israel. This commentary argues that, although postpartum suicidality is relatively rare, it is extremely tragic—not just for the women, but for the entire family and community. The main aim of this commentary is to emphasize that preventive efforts should continue and expand, especially among at-risk groups. At-risk groups include the youngest age group, postpartum Arab women, and postpartum former Soviet Union immigrants. Identification of women at risk or suffering from postpartum depression (PPD) is mandated in Israel. Efforts should include broader screening for various types of suicide ideation and behavior. Assessments should specifically include passive suicide ideation, active suicide ideation with method, intent, and plan, as well as various types of suicide attempts and preparatory behaviors. In addition, specific interventions formulated on evidence-based psychotherapies should be provided in family practice, obstetric, and pediatric settings. These settings are less stigmatized in comparison to mental health settings. Potential therapies can be (among others) Cognitive Behavioral Therapy (CBT) and Interpersonal Psychotherapy (IPT), which are effective in preventing perinatal depression.

## Commentary

In their paper on postpartum suicidality in Israel, Glasser and colleagues [1] review rates, trends and characteristics of postpartum women who considered, attempted, or completed suicide in Israel. Data on suicidal ideation, intentional self-harming behavior, suicide attempts and suicides in pregnant and postpartum women has not been sufficiently collected. This is the first study of its kind in Israel, and a major strength of the study is its use of nation-wide data gathered over an extended period of time. The case example which opens the manuscript is a striking example of the resistance to and stigma about women’s mental health in Israel. Unfortunately, many of the women referred for psychotherapy and/or who are prescribed medication refuse to follow these recommendations; and the social support system around them often is helpless. I applaud the authors since papers like this increase awareness and, I hope, encourage others to continue to study and address the problem.

Postpartum suicidality in Israel was found to be relatively rare; it is both less common than the rate found in non-postpartum Israeli women and, furthermore, it is low relative to the rates found in other countries. We must note, however, that the suicidal behavior which does occur is a significant tragedy for the individual woman, her loved ones and the whole community. Efforts should be made to reduce postpartum suicide to zero, following the international zero tolerance campaigns [2]. Despite the fact that the suicide rate will probably never reach zero, we must continue to aim for that as our goal. Besides the tragedy of the women herself, psychological distress which includes suicide ideation and attempts during pregnancy is a significant risk factor for a range of adverse emotional, cognitive, interpersonal and behavioral outcomes among the child. Maternal suicidal risk may in some cases also lead to parenting patterns which have a damaging impact on child development, the infant-parent attachment and on the mother’s and father’s subsequent mental health [3].

The risk was found to be highest among mothers in the youngest age group and in Arab postpartum women and postpartum former Soviet Union immigrants. The stress related to belonging to a minority group and inequalities in health service utilization between the Jewish and Arab/immigrant sectors, may explain the higher risk found in the two last groups. A study on suicide and suicide attempts in the Arab population in Israel indicated the same trend [4]. Studies have identified a number of practical, psychological, and cultural barriers to mental health service use including cost, inconvenient clinic locations, transportation, limited hours, childcare, stigma, discrimination, previous negative treatment experiences, and the provider’s cultural insensitivity [5]. These should all be specifically assessed and targeted among postpartum women, specifically those in the at-risk groups.

Suicide among prenatal women is related to high levels of psychopathology [6]. Israel can be proud of its Ministry of Health which since 2013 has mandated a program for early identification of women at risk or suffering from postpartum depression (PPD) by nurses in the Mother-and-Child Healthcare Centers. As recommended internationally, the PPD identification program includes three elements: universal screening (using the Edinburgh Postnatal Depression Scale), followed by nurses’ non-directive, supportive counselling intervention, and referral to mental health services for diagnosis and treatment as necessary. Other risk factors should also be targeted directly including other psychiatric disorders (e.g., PTSD, substance use), past suicide attempts, non-suicidal self-injurious behaviors and so on. In addition, it is crucial that protective factors which reduce suicide risk should be targeted. These factors include increased social support, and continued care for both the mother, her partner and her unborn child. What remains an important challenge is the follow-up these women are given and their continued chain of care. Following the example provided by the authors, we should be able to help women and their families comply with professional recommendations.

I think there are four major take home messages from the study. The first is the importance of continuing efforts to increase awareness, assessment and intervention for suicide prevention during and after pregnancy. One can assume that these have contributed to the lower rates of postpartum suicidal ideation reported in more recent years and the relatively stable rate of suicide attempts among Israeli postpartum women between 2006 and 2015.

The second take home message is to identify those from high-risk groups for suicide attempts (youngest age group, Arab postpartum women and FSU immigrants) and other high-risk groups and to (promptly) treat them. Treatment can be provided initially in family practice and obstetric and pediatric settings [7]. The stigma in these settings is lower compared to that found in mental health settings. Mental health professionals in these services should be trained to provide pharmacotherapy and non-directive, supportive counselling which is based on evidence-based psychotherapies [8]. This is in line with the US Preventive Services Task Force [9] which found convincing evidence that counseling interventions such as Cognitive Behavioral Therapy (CBT) and Interpersonal Psychotherapy (IPT) are effective in preventing perinatal depression. IPT, for example, directly focuses on depression and interpersonal factors relevant for the perinatal period (life transitions, grief and loss, interpersonal disputes) [10, 11]. Implementing these therapeutic interventions after the universal screening of pregnant and postpartum women may save lives. It is important to start therapy as early as possible. If needed, the intervention can include an initial engagement session based on the principles of motivational interviewing, which is designed to explore and resolve potential barriers to treatment seeking [12].

Third, it is extremely important that an audit or psychiatric autopsy be conducted on each case of postpartum suicide, as has been done by the Israeli Ministry of Education in the case of students [13]. The psychiatric autopsy is a scientific method for reconstructing a death by suicide through interviews with survivors and examination of all relevant information. The autopsy involves examining the physical and environmental details of the daily life of the deceased to more precisely determine the manner of death and role of the victim in accelerating or influencing his or her own death [14]. Psychological autopsy should be a standard response following a suicide which could make each of these tragic events useful in the prevention of future suicides.

Lastly, the information which is systematically collected on suicide ideation (SI), suicide attempts and suicide among these at-risk women should be reconsidered. When there is indication for risk, there are guidelines for the provider. However, the question about suicide ideation is currently: “In the past week the thought of harming myself has occurred to me: quite often/sometimes/hardly ever/never”. Suicidal ideation is more frequent in pregnant women than in the general population and often presents with higher intent [15]. Therefore, I would encourage broadening the suicide ideation spectrum to include direct questions on passive suicide ideation (thoughts about death), active suicide ideation, method, intent and plans. The various types of suicide ideation may be more prevalent among this population. Similarly, data on self-harm behavior should also be broadened to include aborted suicide attempts, interrupted suicide attempts, preparatory behaviors and non-suicidal self-injurious behaviors [16]. As for future studies, data on completed suicides of women aged 18–44 (which were taken from the national database of causes of death, maintained by the CBS, based on death certificates) should probably be reclassified to make sure there are no cases in which injury or undetermined intent masked suicide [17]. In addition, it is crucial to include psychiatric hospitals in future studies which were not included in the current one. It is likely that the true number of attempts/suicides is somewhat higher when the psychiatric hospitals are included.

## Conclusion

Postpartum suicidality in Israel was found to be relatively low but we should aim to reduce it even more. It is crucial to continue the efforts to prevent postpartum suicide by increasing awareness, assessment and intervention. It is specifically important to identify those from high-risk groups and intervene early. A psychiatric autopsy should be conducted on postpartum suicides so at least we may be able to prevent additional future tragedies.

## Data Availability

Not applicable.

## Abbreviations

CBS: Central Bureau of Statistics (Israel)
CBT: Cognitive behavioral therapy
FSU: Former soviet union
IPT: Interpersonal psychotherapy
PPD: Postpartum depression
PTSD: Post-traumatic stress disorder
SI: Suicide ideation
